# A New Solid Bandage

**Published:** 1863-02

**Authors:** 


A NEW SOLID BANDAGE has been recently contrived by Dr. Hamon. The material is gelatine, or common glue, to which, after being dissolved in water, a portion of alcohol is added. The limb is then padded, in parts where considered desirable, and a bandage applied. This bandage is then brushed over with the solution, and allowed to dry; the process being repeated until sufficient thickness is obtained. If then cut open at the side, a stiff but sufficiently elastic sheath is formed, which fits the limb, and can be removed and replaced at pleasure.—*Brit. Med. Jour.*

